# Complete Genome Sequences of Virus Strains Isolated from Bottle A of the South African Live Attenuated Bluetongue Virus Vaccine

**DOI:** 10.1128/MRA.00310-20

**Published:** 2020-05-28

**Authors:** Peter Coetzee, Alan J. Guthrie, Karen Ebersohn, James N. Maclachlan, Arshad Ismail, Antoinette van Schalkwyk, Estelle H. Venter

**Affiliations:** a Clinical HIV Research Unit, Faculty of Health Sciences, University of the Witwatersrand, Johannesburg, South Africa; b Equine Research Centre, Faculty of Veterinary Science, University of Pretoria, Onderstepoort, Pretoria, South Africa; c Department of Veterinary Tropical Diseases, Faculty of Veterinary Science, University of Pretoria, Onderstepoort, Pretoria, South Africa; d Department of Pathology, Microbiology, and Immunology, School of Veterinary Medicine, University of California, Davis, California, USA; e Sequencing Core Facility, National Institute for Communicable Diseases, Sandringham, Johannesburg, South Africa; f Agricultural Research Council, Department of Vaccine and Diagnostic Development, Onderstepoort, Pretoria, South Africa; g College of Public Health, Medical and Veterinary Sciences, James Cook University, Townsville, Queensland, Australia; KU Leuven

## Abstract

This is a report of the complete genome sequences of plaque-selected isolates of five virus strains included in bottle A of the South African Onderstepoort Biological Products commercial live attenuated bluetongue virus vaccine.

## ANNOUNCEMENT

Bluetongue is an arthropod-borne disease of domestic ruminants, certain wild ungulates, and camelids which is caused by the bluetongue virus (BTV; genus *Orbivirus*, family *Reoviridae*). The BTV genome is composed of 10 segments of double-stranded RNA that cumulatively encode 7 structural (VP1 to VP7) and 4 nonstructural (NS1 to NS4) proteins. Segment 2 of the viral genome is highly variable and encodes the outer coat protein VP2. Currently, 28 distinct serotypes of the virus have been identified based on the interaction of the VP2 protein and neutralizing antibodies that are produced by the host immune system (1). Bluetongue virus is endemic in South Africa, with most of the described serotypes having been isolated from this country. To protect against circulating serotypes, a live pentavalent vaccine consisting of 3 bottles, each containing 5 serotypes per bottle (bottle A, serotypes 1, 4, 6, 12, and 14; bottle B, serotypes 3, 8, 9, 10, and 11; bottle C, serotypes 2, 5, 7, 13, and 17), is produced by Onderstepoort Biological Products (OBP; product no. 2013; registration no. G 0358 [Act 36/1947]) (2). We previously reported the full-genome sequences of the plaque-purified strains contained in bottle C of the OBP vaccine (3). Here, we report the genome sequences of the plaque-purified strains of bottle A of the OBP vaccine.

The serotypes were isolated from bottle A (OBP batch no. 115; expiration date, 11 October 2015) using plaque selection on Vero cells as previously described (4). Prior to RNA extraction, the plaque-selected viruses were propagated for 1 round on Vero cells (25 cm^2^), and the cells were harvested when the cytopathic effect was advanced. Total RNA was extracted from the infected cell pellets using TRIzol (Invitrogen), according to the manufacturer’s instructions. Single-stranded RNA (ssRNA) was subsequently precipitated from the total RNA using LiCl precipitation (2 M final concentration) and collected by centrifugation. The double-stranded RNA (dsRNA) remaining in the supernatant was purified through a QIAquick PCR purification column (Qiagen), and the eluted dsRNA was assessed for concentration and purity using a NanoDrop instrument (Thermo Scientific). The purified dsRNA was amplified using the sequence-independent amplification method described by Potgieter et al. (5). The method involves the ligation of scorpion primers to the ends of the viral genome segments, followed by the conversion of denatured dsRNA to DNA using reverse transcription. The genome is then amplified using primers that are complementary to regions on the ligated scorpion primers (5).

Full-genome amplicons were sequenced on a Sequel instrument (Pacific Biosciences [PacBio], Menlo Park, CA, USA) using a commercial service provider (Sequencing Core Facility, National Institute for Communicable Diseases, Sandringham, Johannesburg, South Africa). Briefly, the sequence library was prepared by subjecting PCR-amplified genomes to the PacBio protocol for preparing multiplexed microbial SMRTbell libraries (“Preparing multiplexed microbial libraries using SMRTbell Express template prep kit 2.0”) but without DNA shearing. The library was subsequently sequenced as a pooled equimolar 8-plex library, with an on-plate concentration of 4 pM DNA. Data were recorded using a 10-h movie time and v3.0 chemistry; Sequel Polymerase v3.0 and single-molecule real-time (SMRT) cells v3 were used. Circular consensus sequencing (CCS) analysis was performed using the PacBio SMRT link v6 software, using default parameters.

High-quality CCS (Q20) reads were generated for all five genomes and ranged from 21,257 to 31,452 reads per genome, with the mean read lengths ranging from 1,711 to 2,161 bp. The final genome sequences were generated from the CCS reads by mapping the data to sequences of South African reference strains of BTV that are available in GenBank, using Geneious Prime v2020.1.1. Consensus sequences were next generated from the mapped CCS reads by majority vote, and the genome segments were trimmed so that they were of the same length as the reference genome segments to which the CCS reads were mapped. In order to ensure that we reported full-length genome nucleotide sequences that included 5′- and 3′-untranslated-region (UTR) sequences, we manually confirmed the presence of terminal conserved hexanucleotide sequences (5′ [GTTAAA] and 3′ [ACTTAC]) that have previously been described as being present on the ends of BTV genome segments (6). The assembled genome segments and the raw sequence reads (FASTQ files) have been submitted to GenBank (accession no. PRJNA401524), with the relevant accession numbers being provided in Table 1.

**TABLE 1:** Characteristics and genome accession numbers of the constituent strains of bottle A of the OBP BTV vaccine

OBP vaccine strain	Genome size (bp)	GenBank accession no. for gene:										SRA accession no.	G+C content (%)
		VP1	VP2	VP3	VP4	VP5	VP6	VP7	NS1	NS2	NS3		
BTV-1	19,430	MT070929	MT070930	MT070931	MT070932	MT070934	MT070937	MT070935	MT070933	MT070936	MT070938	SRR11413045	44.0
BTV-4	19,442	MT070919	MT070920	MT070921	MT070922	MT070924	MT070927	MT070925	MT070923	MT070926	MT070928	SRR11413044	44.2
BTV-6	19,414	MT070909	MT070910	MT070911	MT070912	MT070914	MT070917	MT070915	MT070913	MT070916	MT070918	SRR11413043	44.0
BTV-12	19,404	MT070899	MT070900	MT070901	MT070902	MT070904	MT070907	MT070905	MT070903	MT070906	MT070908	SRR11413042	44.0
BTV-14	19,414	MT070889	MT070890	MT070891	MT070892	MT070894	MT070897	MT070895	MT070893	MT070896	MT070898	SRR11413041	44.2

The newly sequenced vaccine strains were compared against previously sequenced reference strains of BTV (Howell reference strains, Agricultural Research Council—Onderstepoort Veterinary Research Institute [ARC-OVR]), as well as OBP BTV vaccine strains that were sequenced in other studies. Multiple sequence alignments (MUSCLE) and pairwise sequence comparisons were conducted using Geneious Prime v2020.1.1. In general, we found high nucleotide sequence identity between the genomes that were sequenced in this study and previously published OBP BTV vaccine strains and ARC-OVR reference strains that are available in GenBank. The genome of BTV-1 was 99.98% identical to an independently sequenced BTV-1 vaccine strain (RSAvvvv/01; GenBank accession no. KP820897, KP821017, KP821139, KP821259, KP821379, KP821499, KP821621, KP821741, KP821862, and KP821982) and 99.91% identical to the ARC-OVR reference strain of BTV-1 (BTV-1 isolate 5012; accession no. JX272379 through JX272388). The BTV-4 genome was 99.97% identical to a previously sequenced BTV-4 vaccine strain [BTV4-RSA(vacc); accession no. JN255942 through JN255951] and 99.86% identical to the ARC-OVR reference strain of BTV-4 (BTV-4 isolate 79043; accession no. JX272579 through JX272588). The BTV-6 vaccine strain shared 99.98% identity with a previously published BTV-6 vaccine strain (BTV-6 5011-60E; accession no. GQ506506 through GQ506515) and 99.84% identity to the ARC-OVR reference strain of BTV-6 (BTV-6 RSArrrr/06; accession no. GQ506498 through GQ506505, AJ585127, and AJ586703). The BTV-12 strain was 99.86% identical to the ARC-OVR BTV-12 reference strain (BTV-12 isolate 75005; accession no. JX272499 through JX272508), whereas BTV-14 shared 99.84% identity with the ARC-OVR BTV-14 reference strain (BTV-14 isolate BT87/59; accession no. JX272479 through JX272488).

The use of the OBP live attenuated BTV vaccine has been associated with the spread of the vaccine viruses in the field and the exchange of genetic material between wild-type and vaccine strains through genetic reassortment and intragenic recombination. It has been suggested that the exchange of genetic material between vaccine and wild-type viruses may potentially confer a fitness advantage to progeny virions (7, 8). It is therefore of interest to trace the spread of the OBP vaccine strains and their genome segments in the field. The availability of authentic full-genome sequence data of strains that are included in bottle A of the OBP BTV vaccine currently being sold will assist with tracking the spread of the constituent strains in the field.

### Data availability.

The accession numbers for the assembled genome segments and their associated raw sequence reads (SRA accession numbers) are provided in Table 1.

## References

[1] Bumbarov V, Golender N, Jenckel M, Wernike K, Beer M, Khinich E, Zalesky O, Erster O 2020 Characterization of bluetongue virus serotype 28. Transbound Emerg Dis 67:171–182. doi:10.1111/tbed.13338. 31469936

[2] Coetzee P, Stokstad M, Venter EH, Myrmel M, Van Vuuren M 2012 Bluetongue: a historical and epidemiological perspective with the emphasis on South Africa. Virol J 9:198. doi:10.1186/1743-422X-9-198. 22973992 PMC3492172

[3] van den Bergh C, Coetzee P, Guthrie AJ, le Grange M, Venter EH 2016 Complete genome sequences of five bluetongue virus (BTV) vaccine strains from a commercial live attenuated vaccine, a BTV-4 field strain from South Africa, and a reassortant strain isolated from experimentally vaccinated cattle. Genome Announc 4:e00462-16. doi:10.1128/genomeA.00462-16. 27340051 PMC4919390

[4] Howell PG, Kümm NA, Botha MJ 1970 The application of improved techniques to the identification of strains of bluetongue virus. Onderstepoort J Vet Res 37:59–66. 4340799

[5] Potgieter AC, Page NA, Liebenberg J, Wright IM, Landt O, van Dijk AA 2009 Improved strategies for sequence-independent amplification and sequencing of viral double-stranded RNA genomes. J Gen Virol 90:1423–1432. doi:10.1099/vir.0.009381-0. 19264638

[6] Attoui H, Mertens PPC, Becnel J, Belaganahalli S, Bergoin M, Brussaard CP, Chappell JD, Ciarlet M, del Vas M, Dermody TS 2011 The double stranded RNA viruses—Orbivirus, Reoviridae, p 592–603. *In* King AMQ, Adams MJ, Carstens EB, Lefkowitz EJ (ed), Virus taxonomy: ninth report of the International Committee on Taxonomy of Viruses. Elsevier Academic Press, San Diego, CA.

[7] He C-Q, Ding N-Z, He M, Li S-N, Wang X-M, He H-B, Liu X-F, Guo H-S 2010 Intragenic recombination as a mechanism of genetic diversity in bluetongue virus. J Virol 84:11487–11495. doi:10.1128/JVI.00889-10. 20702614 PMC2953192

[8] Nomikou K, Hughes J, Wash R, Kellam P, Breard E, Zientara S, Palmarini M, Biek R, Mertens P 2015 Widespread reassortment shapes the evolution and epidemiology of bluetongue virus following European invasion. PLoS Pathog 11:e1005056. doi:10.1371/journal.ppat.1005056. 26252219 PMC4529188

